# Endogenous assessment of chronic myocardial infarction with T_1ρ_-mapping in patients

**DOI:** 10.1186/1532-429X-17-S1-Q128

**Published:** 2015-02-03

**Authors:** Joep van Oorschot, Hamza El Aidi, Pieter Doevendans, Peter R Luijten, Tim Leiner, Jaco J Zwanenburg

**Affiliations:** 1 Radiology, University Medical Center Utrecht, Utrecht, Netherlands; 2 Cardiology, University Medical Center Utrecht, Utrecht, Netherlands

## Background

Detection of cardiac fibrosis based on endogenous MR characteristics of the myocardium would yield a measurement that can provide quantitative information, is independent of contrast agent concentration, renal function and timing (van Oorschot et al. 2014). In *ex vivo* myocardial infarction (MI) tissue, it has been shown that a significantly higher T_1ρ_ is found in the MI region, and studies in animal models of chronic MI showed the first *in vivo* evidence for the ability to detect myocardial fibrosis with native T_1ρ_-mapping (Witschey et al. 2012; Musthafa et al. 2012). In this study we aimed to translate and validate T_1ρ_-mapping for endogenous detection of chronic MI in patients.

## Methods

### Patients

21 patients (19 M, 2 F, age 55 ± 9 years) underwent an MRI exam 2 to 12 months after clinically confirmed myocardial infarction. The study was performed on a Philips Achieva 1.5 T MR scanner (Philips Healthcare), using a 5-channel cardiac receive coil. Written informed consent was obtained from all patients.

*In vivo MR:* T_1ρ_-mapping was performed using a T_1ρ_-prepared balanced SSFP sequence. 4 images with different spin-lock preparation times (SL) with an amplitude of 750 Hz were acquired (SL = 1,13,27,45 ms). Bandwidth/pixel = 530 Hz, TE/TR = 1.94/3.9 ms, resolution = 1.5x1.65 mm, slice thickness = 6 mm, FOV = 288x288 mm^2^, flip angle = 50 degrees, 2 TFE shots, NSA = 2, SENSE = 1.5. Images were acquired in late diastole during expiration breath holds, with an R-R interval of 3 beats. LGE MRI was performed 15 minutes after contrast injection (0.2 ml/kg contrast agent (Gadovist). (TI = 300-340 ms, TE/TR = 3.5/7.1 ms, resolution = 1.5x1.65 mm, slice thickness = 6 mm, FOV = 288x288 mm^2^, flip angle = 25 degrees, 5 shots).

### Analysis

T_1ρ_-maps were calculated by pixelwise fitting of a mono-exponential decay function in Matlab (Mathworks). The LGE images and T_1ρ_ maps were scored using the 17 segments AHA-model.

## Results

**Figure 1:**
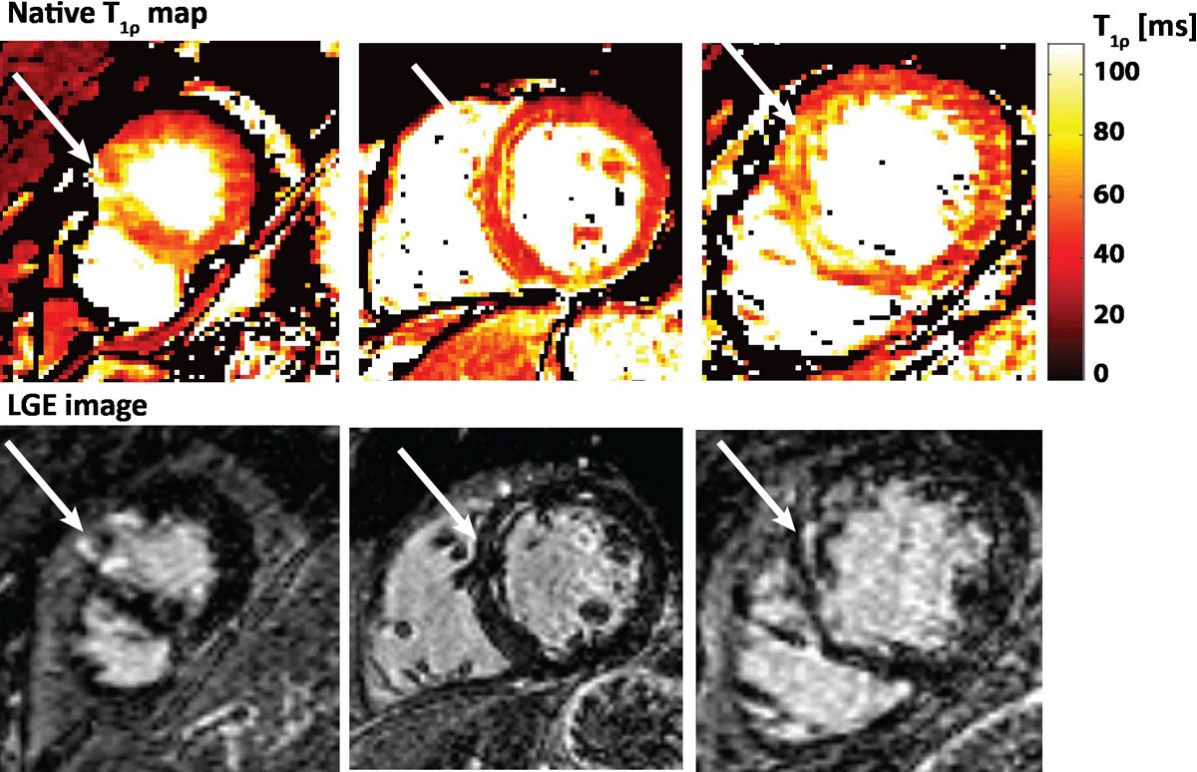
Short axis T_1ρ_-maps with corresponding LGE images in 3 different patients. Arrows indicate the infarcted area.

T_1ρ_ relaxation time was significantly higher in the infarct region (79 ± 11 ms), compared to healthy remote myocardium (54 ± 6 ms), p<0.0005. The myocardial region with an elevated T_1ρ_ relaxation time closely correlated with the corresponding LGE results (figure 1). Overlap in the scoring of scar tissue on LGE images and T_1ρ_-maps was 74 % (table 1).

**Table 1:** Score LGE versus T1ρ in patients with chronic MI (n=21), using the 17 segments AHA-model.

Nr segments:	LGE positive	LGE negative	
T1ρ positive	71	63	0.53 (positive predictive value)

T1ρ negative	22	170	0.89 (negative predictive value)

	0.76 (sensitivity)	0.73 (specificity)	

## Conclusions

We have shown the feasibility of native T_1ρ_-mapping for detection of infarct area in patients with a chronic myocardial infarction. Since this method is quantitative, requires no contrast agent, and thus is independent on renal function and timing, it may provide additional information or be an alternative to the LGE method in patients with severe renal failure.

## Funding

N/A.

